# Long-term effects intensive medical therapy on the development and progression of subclinical atherosclerosis and the metabolic syndrome in Chinese patients with type 2 diabetes mellitus

**DOI:** 10.1097/MD.0000000000005201

**Published:** 2016-11-18

**Authors:** Zhiwen Liu, Zhiguang Zhou, Gan Huang, Yang Xiao, Zhen Li, Cong Liu, Risu Na

**Affiliations:** a Department of Endocrinology, Xuhui District Central Hospital, Shanghai; b Department of Endocrinology; c B-Ultrasound Room, Second Xiangya Hospital of Central South University, Changsha, China.

**Keywords:** metabolic syndrome, subclinical atherosclerosis, type 2 diabetes mellitu

## Abstract

**Background:**

Few studies have investigated the progression of subclinical atherosclerosis and metabolic syndrome (MetS) in Chinese patients with type 2 diabetes mellitus (T2DM). This study was to compare the long-term effects of intensive medical therapy on the development and progression of subclinical atherosclerosis and MetS in Chinese T2DM patients with that of a conventional treatment regimen.

**Methods:**

A total of 316 T2DM patients were randomized to receive conventional pharmacological treatment or intensive medical therapy, consisting of diet and exercise counseling, from 2002 to 2014 at our hospital in Changsha, China. Clinical indicators of subclinical atherosclerosis and MetS were evaluated over the 12-year follow-up period. A *χ*^2^ analysis or *t* tests was used to compare the data between the 2 groups. Risk factors for subclinical atherosclerosis were identified using Cox proportional hazard models.

**Results:**

The incidence of subclinical atherosclerosis increased in both groups over time, and did not differ significantly between the 2 groups at the end of the study. However, after 6 years of treatment, the risk of subclinical atherosclerosis was significantly lower in the intensive medical therapy group, based on intima-media thickness (IMT) measurements, compared with that in the conventional treatment (44.2% vs. 69.7%; *P* < 0.01). Age, creatinine, and IMT of the common iliac artery were significantly associated with subclinical atherosclerosis. Although the indicators of MetS did not differ significantly at the end of study, the success rate for the management of MetS in the intensive medical therapy group was significantly higher than that in the conventional treatment group in 2006, 2008, 2010, and 2012.

**Conclusions:**

The incidence of atherosclerosis in the intensive medical therapy group was significantly lower than that in the conventional treatment group from 2006 to 2010 (*P* < 0.05), and the incidence of MetS in the intensive medical therapy group was significantly higher than that in the conventional treatment group from 2006 to 2012. Kaplan–Meier estimations showed that the risk of subclinical atherosclerosis in the intensive medical therapy group was significantly lower than that in the conventional treatment group (*P* < 0.001), whereas the risk of MetS was not significantly different between the treatment groups (*P* > 0.05).

## 1 Introduction

In recent decades, type 2 diabetes mellitus (T2DM) has become a major public health challenge in developed countries.^[1]^ According to the Diabetes Atlas (Sixth Edition) of the International Diabetic Federation, 387 million patients were treated for T2DM in 2014 worldwide, and the prevalence of T2DM is expected to rise to 592 million by 2035. The global burden of T2DM in 2013 was estimated to be approximately 548 billion USD.^[2]^ Atherosclerotic disease is a leading cause of morbidity and mortality.^[3]^ Previous studies have shown that T2DM is a major risk factor for atherosclerosis, and that T2DM accelerates the progression of atherosclerosis.^[4]^ The identification of atherosclerosis at subclinical stages would facilitate early intervention, leading to improved prognoses.

Previous studies have also shown that T2DM is a major risk factor for metabolic syndrome (MetS), which is characterized by insulin resistance, glucose intolerance, dyslipidemia, hypertension, and obesity.^[3]^ The National Health and Nutrition Examination Survey reported that the prevalence of MetS in the United States was approximately 34% between 1988 and 1994.^[2]^ The prevalence of MetS has been shown to increase with increasing age,^[5]^ and the interaction between the components of MetS has been shown to contribute to the development of atherosclerosis.^[6]^ Various strategies are currently employed to treat T2DM, including lifestyle modifications and medical interventions. However, relatively few trials have investigated the effects of long-term pharmacological treatment for subclinical atherosclerosis and MetS in T2DM patients. In addition, relatively few studies of subclinical atherosclerosis and MetS have been reported for Asian populations, for whom diet and lifestyle risk factors can differ significantly from those of their Western counterparts.

We performed a randomized, controlled study comparing the efficacy of intensive medical therapy and that of conventional treatment regimens for slowing the development and progression of subclinical atherosclerosis and improving hyperglycemia, hypertension, dyslipidemia, and albuminuria in Chinese T2DM patients. Our findings provide valuable clinical evidence relevant to the development of more effective early intervention strategies for atherosclerosis and MetS-related complications in Chinese T2DM patients.

## 2 Participants and methods

### 2.1 Participants

We enrolled 316 patients who had received a diagnosis of T2DM at the Second Xiangya Hospital of Central South University (Changsha, China) between January 3 and October 31, 2002. The inclusion criteria were as follows: diagnosis of T2DM based on the World Health Organization (1999) diagnostic guidelines; 35 to 70 years of age; disease duration <1 year; and body mass index (BMI) of 19 to 35 kg/m^2^. Patients meeting the following criteria were excluded from our study: diagnosis of type 1 diabetes mellitus; ≥1 episodes of diabetic ketoacidosis or other adverse stress responses within the first 6 months following T2DM diagnosis; a diagnosis of subclinical atherosclerosis at baseline based on a Doppler color ultrasound examination; serum creatinine >2.0 mg/dL; serum ALT >80 U/L; or acute diabetic complications, including heart disease, neuropathy, nephropathy, or peripheral vascular disease. Our study was approved by the institutional review board of our institution, and informed written consent was obtained from all of the patients before their participation in our study.

### 2.2 Data and sample collection

A standardized questionnaire was used to collect information on age, smoking (current, former, or never), alcohol consumption (yes, no, or former), and family history of MetS manifestations, as described previously.^[7]^ Blood samples were collected using EDTA as an anticoagulant. The blood samples were centrifuged, and the plasma was stored at −80°C.

### 2.3 Clinical variables

Height, weight, waist circumference (WC), hip circumference (HC), and blood pressure (BP) were recorded as described previously.^[8]^ Total cholesterol (TC), triglyceride (TG), high-density lipoprotein cholesterol (HDL-C), and low-density lipoprotein cholesterol (LDL-C) were measured using a Hitachi 7170 Automated Clinical Analyzer (Tokyo, Japan) with proprietary reagents. The total bilirubin (TBil) and alanine aminotransferase (ALT) levels were measured using a Toshiba TBA2000FR Automated Biochemical Analyzer (Tokyo, Japan). Blood urea nitrogen (BUN) was measured using a Hitachi 7180 Automated Clinical Analyzer using reagents from Roche Diagnostics (Indianapolis, IN). Urinary creatinine (CR) and 24-h urinary albumin (UAlb) were measured using a picric acid colorimetric method^[9]^ an immunoturbidimetric assay,^[10]^ respectively. The 2-hour postprandial plasma glucose level (2hPG) and overnight fasting plasma glucose level (FPG) were measured using the glucose oxidase method.^[11]^ Glycated hemoglobin (HbA1c) was measured using a Bio-Rad Variant II HPLC System (Hercules, CA) equipped with an ion exchange cartridge. Fasting plasma insulin (FINS) was measured using a chemiluminescent immunoassay (Siemens Healthcare, Erlangen, Germany). The insulin resistance index was evaluated based on the homeostasis model assessment of insulin resistance (HOMA-IR), as described previously,^[12]^ according to the following equation: (FPG [mmol/L]) × (FINS [μU/mL])/22.5. Glomerular filtration rate (GFR) was measured with ^99m^Tc-DTPA renal dynamic imaging using a Millennium TMMPR SPECT (GE Healthcare Life Sciences, Pittsburgh, PA), as described previously,^[13]^ and the GFR was calculated based on the traditional MDRD equation.^[14]^ The IMT of the common carotid artery (CCA), femoral artery (FA), and common iliac artery (CIA) were evaluated as described previously.^[8]^

### 2.4 Diagnostic criteria

Proteinuria was defined as a UAlb concentration >30 mmol/L. Subclinical atherosclerosis was defined as IMT >1.0 mm and/or detectable plaque in the lumen of the CCA, FA, or CIA without clinical manifestations.^[15]^ The diagnostic criteria for MetS were based on those published by the WHO in 1999, which require the presence of T2DM, impaired glucose tolerance, impaired fasting glucose, or insulin resistance and 2 of the following: a systolic blood pressure to diastolic blood pressure ratio (SBP/DBP) ≥140/90 mm Hg; dyslipidemia based on TG ≥1.7 mmol/L and HDL-C ≤0.9 mmol/L (men) or ≤1.0 mmol/L (women); central obesity based on BMI >30 kg/m^2^ or a WC to HC ratio (WHR) >0.9 (men) or >0.85 (women); or microalbuminuria based on a UAlb excretion rate (UAER) ≥20 μg/min or a UAlb to CR ratio (ACR) ≥30 mg/g.

### 2.5 Treatment groups

Patients were randomly assigned to the intensive medical therapy group or the conventional treatment group. Patients in the intensive medical therapy group received diet and exercise counseling from a diabetes educator on a monthly basis. Their FPG, 2hPG, BP, body weight, heart rate, and pulse rate at the dorsal artery of the foot were measured monthly. When hyperglycemia occurred, obese and nonobese patients were first treated with metformin or glipizide, respectively, combined with additional glucose-lowering drugs in some cases. Insulin therapy was prescribed for cases in which metformin or glipizide was ineffective. When hypertension occurred, patients were first treated with captopril or valsartan combined with extended-release nifedipine or other antihypertensive drugs in some cases. When TC or TG levels exceeded the target concentrations, patients were treated with simvastatin or fenofibrate. When albuminuria occurred, patients were treated with captopril or valsartan. Patients also received dietary advice and exercise instructions to assist them in maintaining a near-normal bodyweight.

Patients in the conventional treatment group were treated in the outpatient clinic, and received conventional pharmacological therapy. They underwent a follow-up examination every 3 months from March 2002 to December 2014. Their FPG, 2hPG, BP, body weight, heart rate, and pulse rate at the dorsal artery of the foot were measured every 3 months, and their TC, TG, HDL-C, LDL-C, and HbA1c were measured every 6 months. Once yearly, their UAlb, CR, FINS, height, WC, and HC were measured, and they underwent vascular ultrasound to assess the thickness of the intima-media (IMT) of the CCA, FA, and CIA. The IMT is used routinely in clinical practice to evaluate subclinical atherosclerosis.^[16]^ The target values for the clinical variables were as follows for both treatment groups: HbA1c <6.5%; SBP/DBP <130/80 mmHg; TG <1.7 mmol/L; HDL-C >1.04 mmol/L (men) or >1.3 mmol/L (women); WHR <0.85 (men) or <0.8 (women); UAER <20 μg/min; and BMI <24 kg/m^2^. All of the drugs required in both groups were purchased by the patients, and the costs were reimbursed through the national health insurance program.

### 2.6 Statistical analysis

The statistical analysis was performed using the SAS, version 9.3, software (SAS Institute, Cary, NC). Intergroup differences in the patient characteristics and clinical variables were evaluated using 2-tailed *χ*^2^ tests for categorical variables and 2-tailed Student *t* test for continuous variables. A generalized linear mixed model was used to compare the intergroup differences in IMT values, which controlled for potential covariates, including sex, family history, BMI, BP, and FINS. Results with *P* < 0.05 were considered statistically significant. Univariate and multivariate regressions were performed using Cox proportional hazards models to identify factors associated with incident subclinical atherosclerosis. Significant risk factors (*P* < 0.2) identified in the univariate analysis were included in the multivariate analysis using the Cox proportional hazards model. The hazard ratios (HRs) and 95% confidence intervals (CIs) were calculated, and *P* < 0.05 was considered to indicate a statistically significant association. Kaplan-Meier estimations of the risks of subclinical atherosclerosis and MetS were performed, and the significance of the difference in risk between the treatment groups was determined by the log-rank test.

## 3 Results

### 3.1 Patient characteristics

**Figure 1:**
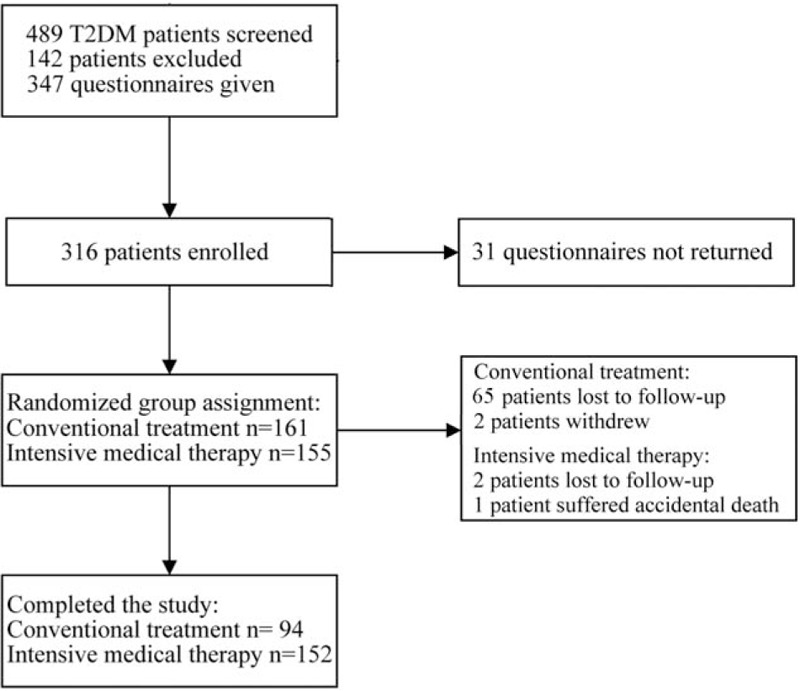
Flow chart depicting patient recruitment and selection, the number of patients who withdrew from the study, and the number of patients who were lost to follow-up.

After screening a total of 489 patients with T2DM, 316 patients were enrolled in our study, with a baseline sample of 155 patients in the intensive medical therapy group and 161 patients in the conventional treatment group. One patient withdrew from the study owing to cerebral infarction, and another withdrew because of bladder cancer. A total of 70 patients were lost to follow-up, leaving a total of 246 patients (77.8%) who completed the study (Fig. 1). Although no significant differences were observed in patient characteristics at baseline, significantly greater percentages of smokers, alcohol users, and patients with a family history of MetS and higher levels of FPG, 2hPG, and LDL were observed in the conventional treatment group at the end of the study period, compared to those in the intensive medical therapy group in 2014 (Table 1).

**Table 1:**
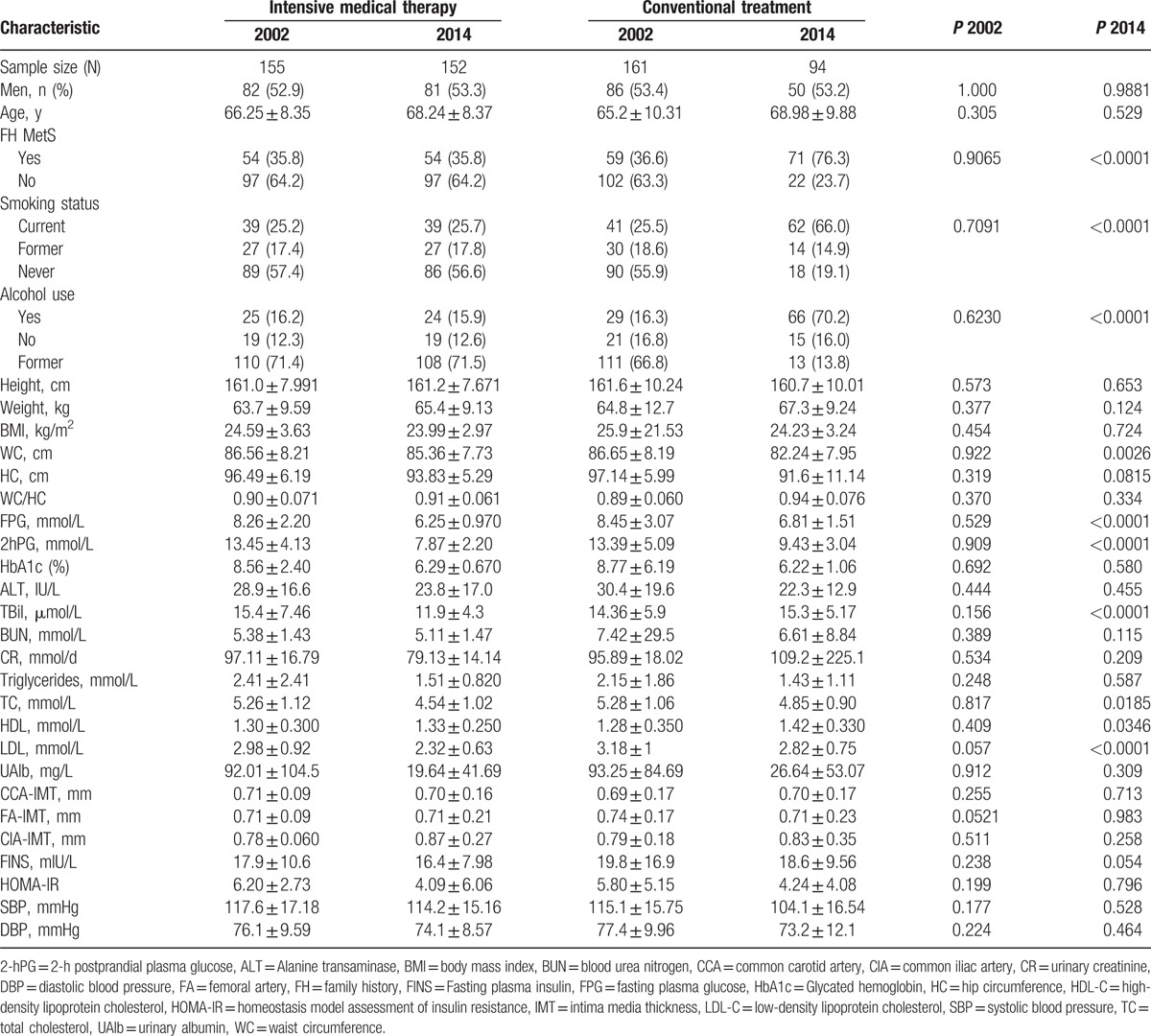
Comparison of patient characteristics at baseline and study endpoint.

### 3.2 Incidence of atherosclerosis

**Figure 2:**
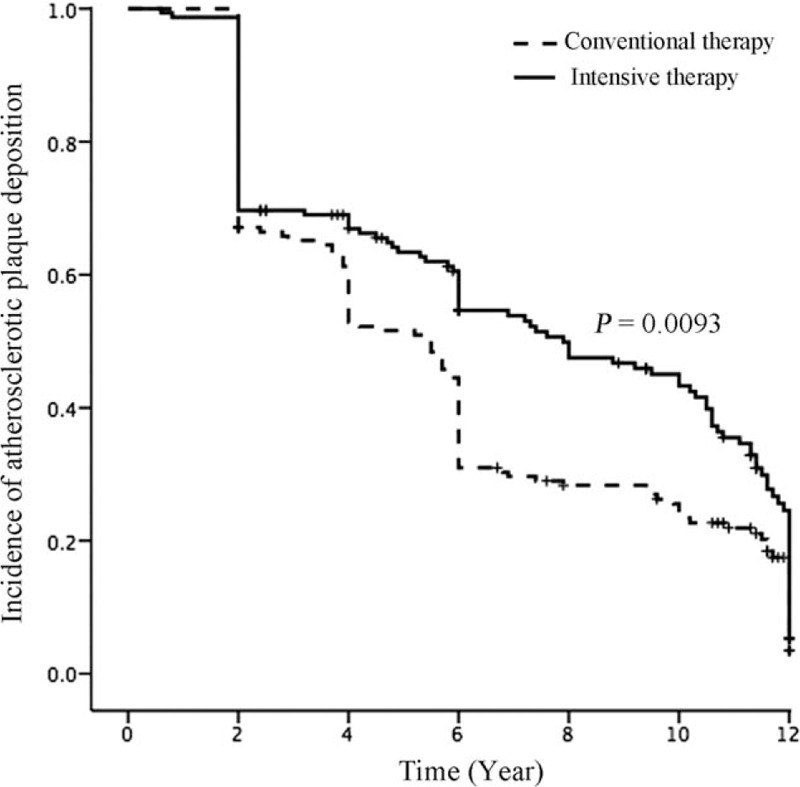
Kaplan–Meier analysis of the differences in the risk of subclinical atherosclerosis between the treatment groups (*P* < 0.001 by the log-rank test).

The incidence of atherosclerosis in the intensive medical therapy group was significantly lower than that in the conventional treatment group from 2006 to 2010 (Table 2). A Kaplan–Meier analysis showed that the risk of developing subclinical atherosclerosis in the conventional treatment group was significantly greater than that in the intensive medical therapy group (Fig. 2; *P* = 0.0093 by log-rank test).

**Table 2:**
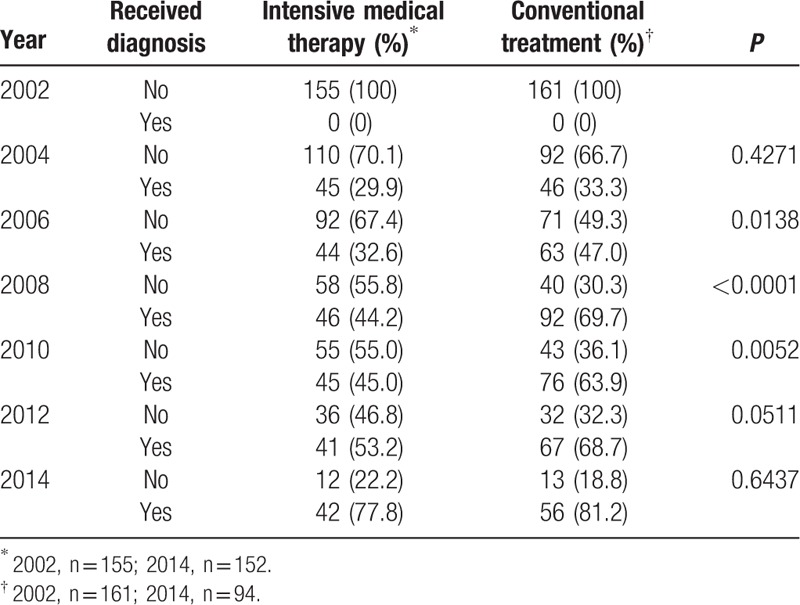
Rates of developing subclinical atherosclerosis over the follow-up period.

### 3.3 Risk factors for atherosclerosis

To identify predictors of subclinical atherosclerosis in T2DM patients, we evaluated whether the various demographic and clinical variables were risk factors for the development of atherosclerotic plaque. We found that age, CR, and CIA-IMT were significantly associated with the incidence of subclinical atherosclerosis (Table 3). The incidence of atherosclerosis increased with increasing age at a rate of approximately 5% per year. The HR of CIA-IMT for the incidence of atherosclerosis was 5 times higher than that of the other risk factors identified (Tables 3 and 4).

**Table 3:**
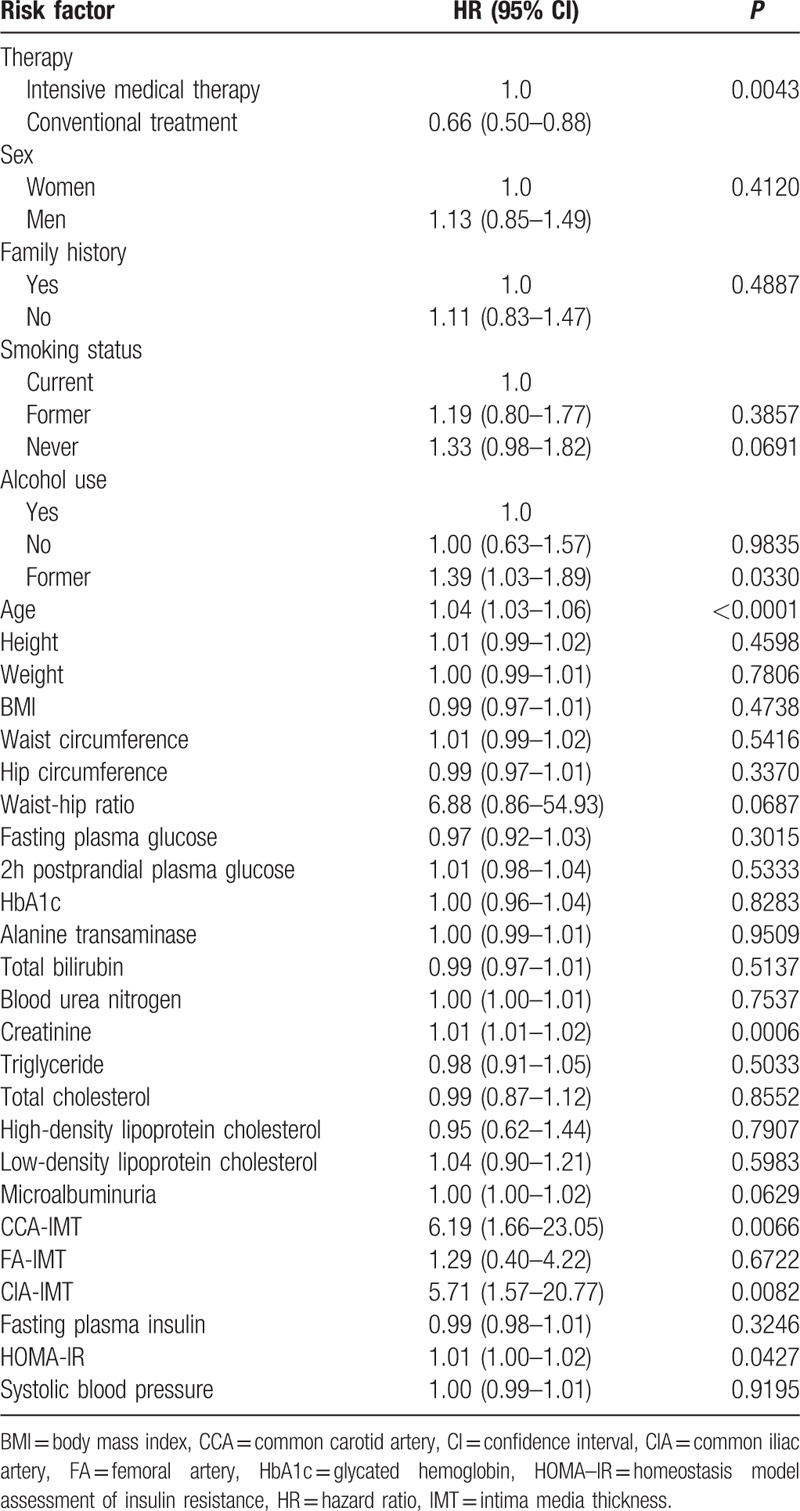
Univariate analysis of risk factors for subclinical atherosclerosis.

**Table 4:**
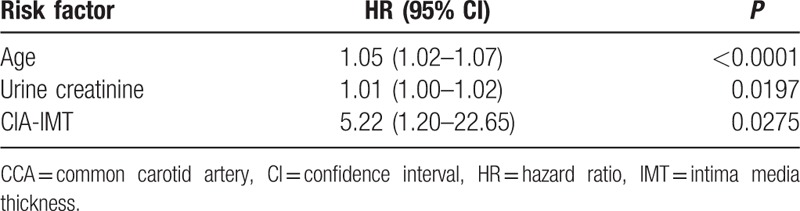
Multivariate analysis of the risk of subclinical atherosclerosis.

### 3.4 Treatment effects on IMT

The IMTs of the CCA, FA, and CIA were significantly lower in the intensive medical therapy group in 2008 than that in the conventional treatment group (Table 5). However, the IMTs of the intensive medical therapy and conventional treatment groups did not differ significantly at the end of the study period. The IMTs of all of the patients in both groups were >1 mm in 2014, indicating that all of the patients had subclinical atherosclerosis at the end of the study period.

**Table 5:**
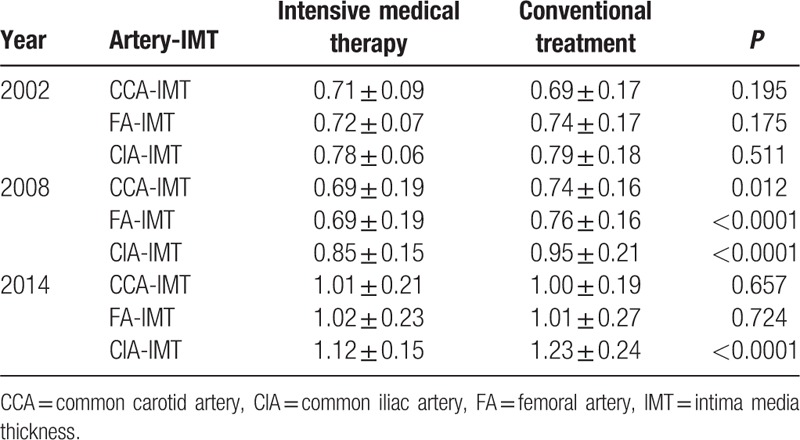
Comparison of atherosclerotic plaque deposition between the treatment groups based on intima media thickness.

### 3.5 Treatments effects on renal function and HOMA-IR

We also analyzed renal function and HOMA-IR as indicators of MetS. The HOMA-IR did not differ significantly between the intensive medical therapy and conventional treatment groups in 2003, 2008, and 2014 (Table 6). However, differences in renal function were observed. Albuminuria in 2003 was significantly lower in the intensive medical therapy group, compared with that of the conventional treatment group, with the BUN, CR, and GFR decreasing in both groups over the 12-year follow-up period (Table 6).

**Table 6:**
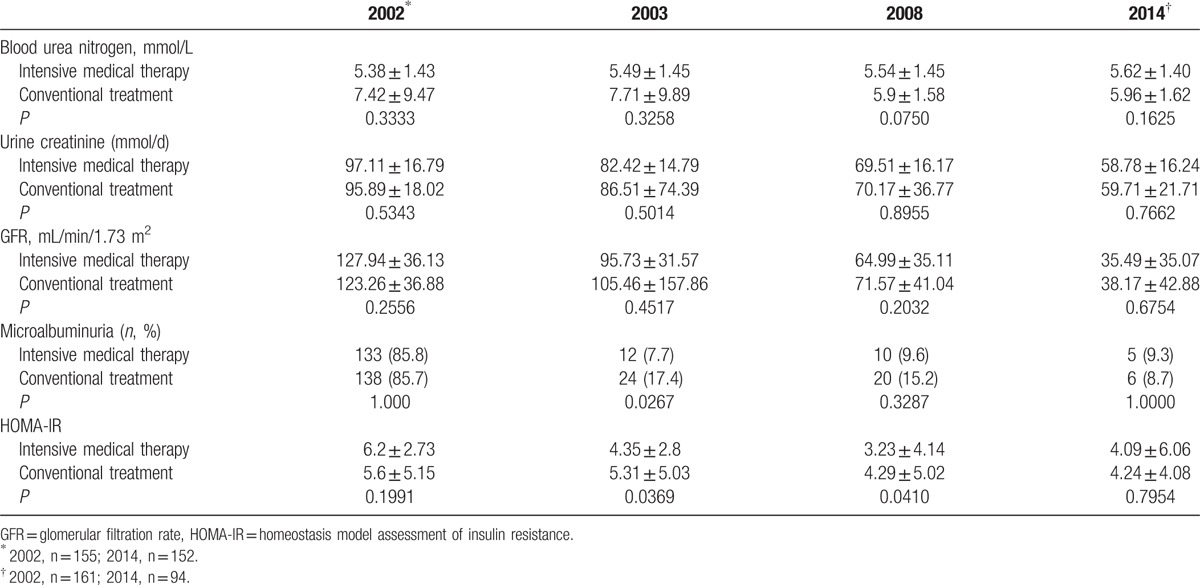
Comparison of renal function indexes and insulin resistance between the treatment groups.

### 3.6 Treatment effectiveness for controlling MetS

**Figure 3:**
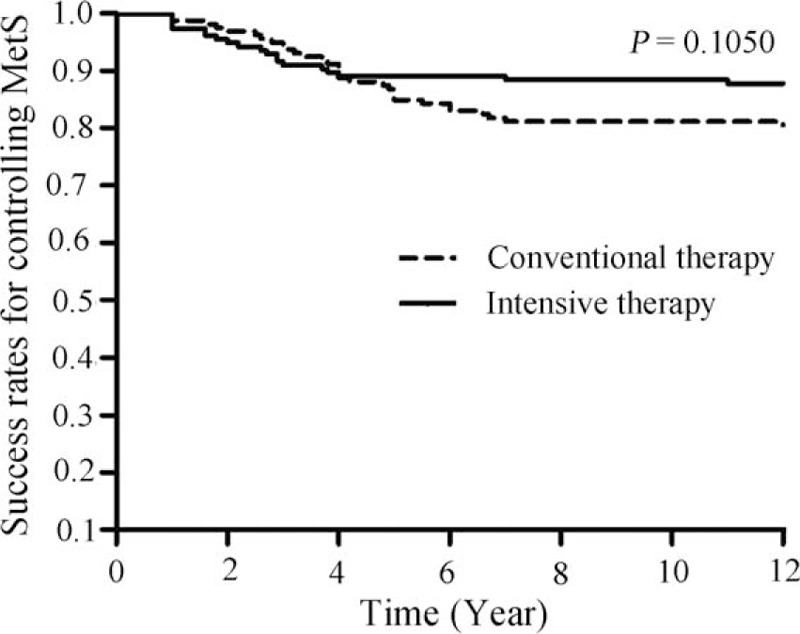
Kaplan–Meier analysis of the differences in the risk of metabolic syndrome between the treatment groups (*P* > 0.05 by the log-rank test).

We examined whether the target values for the HbA1c, BP, lipid profile, WHR, BMI, and microalbuminuria were achieved to assess the effectiveness of the treatments for controlling MetS. The success rate for the management of MetS in the conventional treatment group was significantly higher than that of the intensive medical therapy group from 2006 to 2012 (Table 7). However, the Kaplan-Meier estimation did not show a significant difference in the risk of MetS between the study groups (Fig. 3; *P* = 0.105 by log-rank test).

**Table 7:**
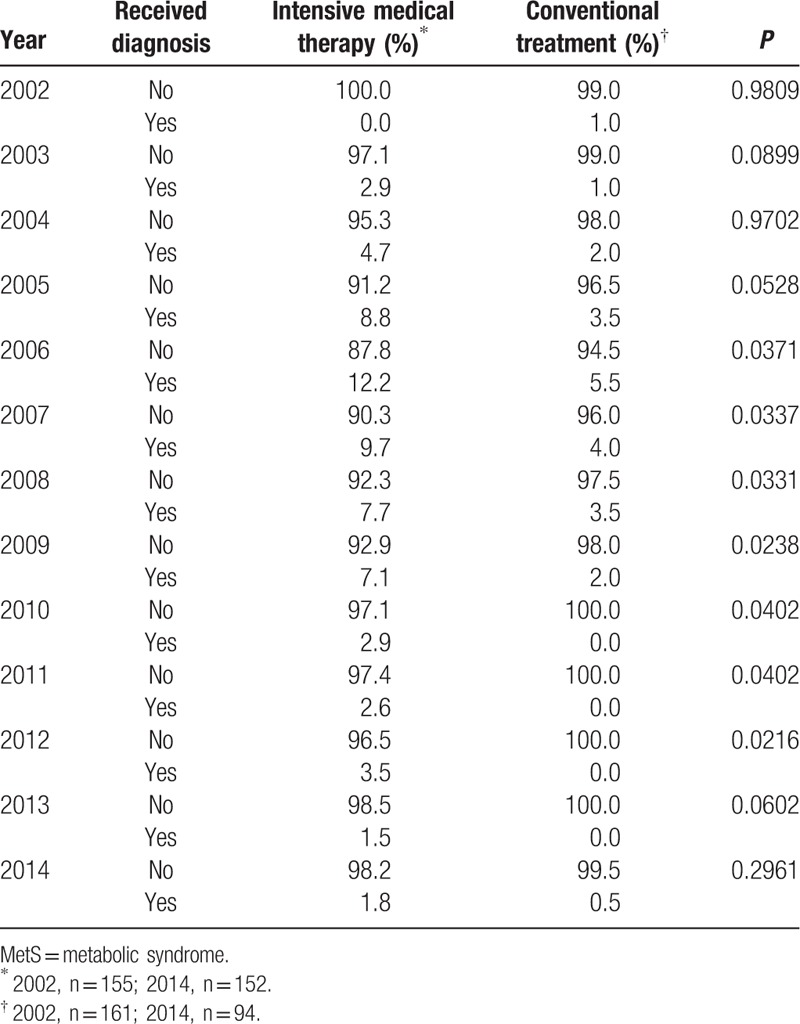
Rates of developing metabolic syndrome over the follow-up period.

## 4 Discussion

We compared the effectiveness of intensive medical therapy for subclinical atherosclerosis and MetS in Chinese T2DM patients with that of conventional treatment. In the intensive medical therapy group, metformin or glipizide was the first choice for lowering blood glucose. Metformin is an oral biguanide drug, and glipizide is an oral, rapidly absorbed, short-acting sulfonylurea drug.^[17]^ Captopril, an angiotensin-converting enzyme inhibitor, or valsartan, an angiotensin II receptor antagonist, was used to control hypertension and albuminuria, both of which have been used to treat hypertension and congestive heart failure.^[18,19]^ Simvastatin or fenofibrate was used to treat dyslipidemia, as described previously.^[20,21]^

Atherosclerosis is a systemic disease affecting multiple layers of the arterial wall. We focused our investigation on subclinical atherosclerosis because it is a risk factor for cardiovascular events.^[15,22]^ The presence of atherosclerotic plaque and IMT are both used as markers of subclinical atherosclerosis. We first compared the incidence of subclinical atherosclerosis between the intensive medical therapy and conventional treatment groups. The incidence of subclinical atherosclerosis increased steadily over time in both groups, and was not significantly different between the treatment groups at the end of the study. However, the incidence of subclinical atherosclerosis in the conventional treatment group was higher than that in the intensive medical therapy group at the 6-year follow-up examination.

Age, CR, and CIA-IMT were identified as significant risk factors for atherosclerosis in both groups, which is consistent with the findings of previous studies.^[23,24]^ We also found that the IMT of the CCA, FA, and CIA of patients in the intensive medical therapy group were significantly lower after 6 years of treatment than those in the conventional treatment group. However, the IMTs did not differ significantly between the 2 groups at the end of the study period. These results showed that intensive medical therapy reduced the incidence of subclinical atherosclerosis from 2006 to 2010 only, but the Kaplan–Meier analysis of the data showed that intensive medical therapy significantly reduced the overall risk of subclinical atherosclerosis, compared with that in the conventional treatment group, suggesting that different treatment strategies might be needed to reduce atherosclerotic deposition in Chinese T2DM patients after 6 years of intensive medical therapy.

Our findings are also consistent with those of previous studies, which showed that the carotid artery IMT increased progressively in T2DM patients treated with metformin for 24 weeks,^[16]^ and that it did not differ significantly between the patients treated with metformin for 18 months and those who received the placebo treatment.^[13]^ The long-term effect of metformin treatment on the risk of cardiovascular disease remains largely unclear.^[20]^ In addition, a previous study showed that, although treatment with valsartan for 24 months did not influence CCA-IMT, it did improve vascular wall function in patients with essential hypertension.^[14]^

The development of subclinical atherosclerosis has also been reported in patients who had been treated with simvastatin.^[25]^ Fenofibrate has been shown to inhibit the progression of carotid IMT in people with essential hypertension and mild hyperlipidemia,^[26]^ but did not significantly inhibit the progression of carotid IMT in T2DM patients.^[27]^ A limited number of studies have investigated the role of glipizide and captopril in preventing subclinical atherosclerosis. In general, the use of these drugs alone is ineffective for preventing subclinical atherosclerosis. However, our results showed that the use of these drugs in combination reduced the risk of subclinical atherosclerosis in Chinese T2DM patients.

We also explored the effect of intensive medical therapy on indicators of MetS, and found that the HOMA-IR index, GFR, albuminuria, and CR decreased in both groups over the 12-year follow-up, suggesting that both therapies were effective for reducing markers of MetS in T2DM patients. The proportion of patients with albuminuria in the intensive medical therapy group was significantly lower in 2003 than that of the conventional treatment group. These results suggest that intensive medical therapy is more effective for maintaining renal function in Chinese T2DM patients, compared with the effects of the conventional treatment.

We also evaluated the effectiveness of intensive medical therapy for the management of MetS, compared to that of the conventional treatment regimen. The success rate for the intensive medical therapy group was significantly lower than that of the conventional treatment group from 2006 to 2012. However, the success rate in the intensive medical therapy group did not differ significantly from that of conventional treatment group in the latter years of the follow-up period, and a Kaplan–Meier analysis did not show a significant difference in MetS risk between the treatment groups. Studies in both animals and humans have suggested that treatment with metformin,^[28]^ captopril,^[29,30]^ valsartan,^[31,32]^ simvastatin,^[33]^ or fenofibrate ^[34,35]^ alone reduced the development of MetS, which is largely consistent with our findings. Because MetS increases the risk of cardiovascular disease,^[36–38]^ it seems unlikely that improvements in MetS indicators contributed to a lower risk of subclinical atherosclerosis in the intensive medical therapy group, compared with that in the conventional treatment group.

The average age of the intensive medical therapy and conventional treatment groups (66 and 65 years at baseline, respectively) may have confounded our results because the risks of MetS and subclinical atherosclerosis have both been shown to increase with increasing age,^[39,40]^ which might have contributed to the lack of a significant difference in incidence rates between the treatment groups in the latter years of the follow-up period. Although all patients were reimbursed for drug costs, the greater initial economic burden for the conventional treatment group may have contributed to a higher drop-out rate. The significantly greater percentages of smokers, alcohol users, and patients with a family history of MetS and higher levels of FPG, 2-hPG, and LDL in the conventional treatment group at the end of the study period, compared to those in the intensive medical therapy group in 2014, might have also influenced our results. However, these differences would seem more likely to contribute to a greater difference between the treatment groups, which was not the case for our analysis.

Although we are unaware of other investigations that have compared the effects of intensive medical therapy and conventional pharmacological treatment on both subclinical atherosclerosis and MetS in T2DM patients in China, the relatively small size of our study population warrants confirmation our findings in a larger cohort of T2DM patients in China. Previous studies have investigated factors contributing to the development of subclinical atherosclerosis and MetS in Chinese Americans.^[41–43]^ However, diet and environmental factors for Chinese people in the United States differ from those of residents of mainland China.^[44]^ Furthermore, variation occurs between different methods of measuring atherosclerotic deposition. Therefore, the use of flow-mediated vasodilatation, arterial stiffness, or the ankle-brachial index for assessing atherosclerotic plaque deposition in future studies is also warranted.

In conclusion, we found that, although Chinese T2DM patients with subclinical atherosclerosis and MetS remained at a high risk for developing more severe atherosclerotic disease after 12 years of intensive medical therapy, the progression of subclinical atherosclerosis and MetS indicators were reduced in Chinese T2DM patients during the first 6 years of intensive medical therapy, compared with those of patients receiving conventional treatment. Our findings provide important clinical data for the improvement of strategies for the prevention and treatment of atherosclerosis and MetS in Chinese T2DM patients.

## References

[1] Danaei G Finucane MM Lu Y National, regional, and global trends in fasting plasma glucose and diabetes prevalence since 1980: systematic analysis of health examination surveys and epidemiological studies with 370 country-years and 2.7 million participants. *Lancet* 2011; 378:31–40. 21705069 10.1016/S0140-6736(11)60679-X

[2] Ford ES Giles WH Dietz WH Prevalence of the metabolic syndrome among US adults: findings from the third National Health and Nutrition Examination Survey. *JAMA* 2002; 287:356–359. 11790215 10.1001/jama.287.3.356

[3] Houschyar KS Ludtke R Dobos GJ Effects of phlebotomy-induced reduction of body iron stores on metabolic syndrome: results from a randomized clinical trial. *BMC Med* 2012; 1054. 10.1186/1741-7015-10-54 PMC3386865 22647517

[4] Chait A Bornfeldt KE Diabetes and atherosclerosis: is there a role for hyperglycemia? *J Lipid Res* 2009; 50 (Suppl):S335–S339. 19029122 10.1194/jlr.R800059-JLR200 PMC2674740

[5] Vishram JK Borglykke A Andreasen AH Impact of age and gender on the prevalence and prognostic importance of the metabolic syndrome and its components in Europeans. The MORGAM Prospective Cohort Project. *PLoS One* 2014; 9:e107294. 25244618 10.1371/journal.pone.0107294 PMC4171109

[6] Kaur J A comprehensive review on metabolic syndrome. *Cardiol Res Pract* 2014; 2014:943162. 24711954 10.1155/2014/943162 PMC3966331

[7] Ye X Yu Z Li H Distributions of C-reactive protein and its association with metabolic syndrome in middle-aged and older Chinese people. *J Am Coll Cardiol* 2007; 49:1798–1805. 17466231 10.1016/j.jacc.2007.01.065

[8] Xiao Y Xu A Hui X Circulating lipocalin-2 and retinol-binding protein 4 are associated with intima-media thickness and subclinical atherosclerosis in patients with type 2 diabetes. *PLoS One* 2013; 8:e66607. 23799122 10.1371/journal.pone.0066607 PMC3684582

[9] Komatsu J Fujiwara M Matsuura M Study on the usefulness of high-molecular-weight (HMW)-Adiponectin level check of Japanese general population upon health check: comparison of carotid ultrasonography measurement. *Clin Biochem* 2012; 45:72–76. 22061336 10.1016/j.clinbiochem.2011.10.013

[10] Gentilini F Dondi F Mastrorilli C Validation of a human immunoturbidimetric assay to measure canine albumin in urine and cerebrospinal fluid. *J Vet Diagn Invest* 2005; 17:179–183. 15825501 10.1177/104063870501700214

[11] Wong CM Wong KH Chen XD Glucose oxidase: natural occurrence, function, properties and industrial applications. *Appl Microbiol Biotechnol* 2008; 78:927–938. 18330562 10.1007/s00253-008-1407-4

[12] Ueda H Ikegami H Kawaguchi Y Age-dependent changes in phenotypes and candidate gene analysis in a polygenic animal model of Type II diabetes mellitus; NSY mouse. *Diabetologia* 2000; 43:932–938. 10952468 10.1007/s001250051472

[13] Liu X Lv L Wang C Comparison of prediction equations to estimate glomerular filtration rate in Chinese patients with chronic kidney disease. *Intern Med J* 2012; 42:e59–e67. 21118405 10.1111/j.1445-5994.2010.02398.x

[14] Levey AS Coresh J Greene T Using standardized serum creatinine values in the modification of diet in renal disease study equation for estimating glomerular filtration rate. *Ann Intern Med* 2006; 145:247–254. 16908915 10.7326/0003-4819-145-4-200608150-00004

[15] Balbarini A Buttitta F Limbruno U Usefulness of carotid intima-media thickness measurement and peripheral B-mode ultrasound scan in the clinical screening of patients with coronary artery disease. *Angiology* 2000; 51:269–279. 10778996 10.1177/000331970005100401

[16] Kotliar C Forcada P Ferdinand KC Noninvasive diagnosis of subclinical atherosclerosis in cardiometabolic syndrome: a call to action. *J Cardiometab Syndr* 2008; 3:60–62. 18326984 10.1111/j.1559-4572.2008.07957.x

[17] Holecek M Three targets of branched-chain amino acid supplementation in the treatment of liver disease. *Nutrition* 2010; 26:482–490. 20071143 10.1016/j.nut.2009.06.027

[18] Yoshiji H Noguchi R Kitade M Branched-chain amino acids suppress insulin-resistance-based hepatocarcinogenesis in obese diabetic rats. *J Gastroenterol* 2009; 44:483–491. 19319465 10.1007/s00535-009-0031-0

[19] Baracos VE Mackenzie ML Investigations of branched-chain amino acids and their metabolites in animal models of cancer. *J Nutr* 2006; 136 (1 Suppl):237S–242S. 16365090 10.1093/jn/136.1.237S

[20] Reizes O Barylko B Li C Domain structure of a mammalian myosin I beta. *Proc Natl Acad Sci U S A* 1994; 91:6349–6353. 8022785 10.1073/pnas.91.14.6349 PMC44199

[21] Togo S Tanaka K Morioka D Usefulness of granular BCAA after hepatectomy for liver cancer complicated with liver cirrhosis. *Nutrition* 2005; 21:480–486. 15811769 10.1016/j.nut.2004.07.017

[22] Kardys I Oei HH van der Meer IM Lipoprotein-associated phospholipase A2 and measures of extracoronary atherosclerosis: the Rotterdam Study. *Arterioscler Thromb Vasc Biol* 2006; 26:631–636. 16373603 10.1161/01.ATV.0000201289.83256.cf

[23] Qian J Maehara A Mintz GS Impact of gender and age on in vivo virtual histology-intravascular ultrasound imaging plaque characterization (from the global Virtual Histology Intravascular Ultrasound [VH-IVUS] registry). *Am J Cardiol* 2009; 103:1210–1214. 19406261 10.1016/j.amjcard.2009.01.031

[24] Gale CR Ashurst H Phillips NJ Renal function, plasma homocysteine and carotid atherosclerosis in elderly people. *Atherosclerosis* 2001; 154:141–146. 11137093 10.1016/s0021-9150(00)00448-2

[25] Hecht HS Harman SM Comparison of the effects of atorvastatin versus simvastatin on subclinical atherosclerosis in primary preventionas determined by electronbeam tomography. *Am J Cardiol* 2003; 91:42–45. 12505569 10.1016/s0002-9149(02)02995-8

[26] Zhu S Su G Meng QH Inhibitory effects of micronized fenofibrate on carotid atherosclerosis in patients with essential hypertension. *Clin Chem* 2006; 52:2036–2042. 16990410 10.1373/clinchem.2006.074724

[27] Hiukka A Westerbacka J Leinonen ES Long-term effects of fenofibrate on carotid intima-media thickness and augmentation index in subjects with type 2 diabetes mellitus. *J Am Coll Cardiol* 2008; 52:2190–2197. 19095138 10.1016/j.jacc.2008.09.049

[28] Orchard TJ Temprosa M Goldberg R The effect of metformin and intensive lifestyle intervention on the metabolic syndrome: the Diabetes Prevention Program randomized trial. *Ann Intern Med* 2005; 142:611–619. 15838067 10.7326/0003-4819-142-8-200504190-00009 PMC2505046

[29] Roncal CA Reungjui S Sanchez-Lozada LG Combination of captopril and allopurinol retards fructose-induced metabolic syndrome. *Am J Nephrol* 2009; 30:399–404. 19696478 10.1159/000235731 PMC2783362

[30] Ernsberger P Johnson JL Rosenthal T Therapeutic actions of allylmercaptocaptopril and captopril in a rat model of metabolic syndrome. *Am J Hypertens* 2007; 20:866–874. 17679035 10.1016/j.amjhyper.2007.02.015 PMC2930912

[31] Shishido T Konta T Nishiyama S Suppressive effects of valsartan on microalbuminuria and CRP in patients with metabolic syndrome (Val-Mets). *Clin Exp Hypertens* 2011; 33:117–123. 21269062 10.3109/10641963.2010.531837

[32] Miyata M Ikeda Y Nakamura S Effects of valsartan on fibrinolysis in hypertensive patients with metabolic syndrome. *Circ J* 2012; 76:843–851. 22451451 10.1253/circj.cj-12-0153

[33] Hunninghake DB Ballantyne CM Maccubbin DL Comparative effects of simvastatin and atorvastatin in hypercholesterolemic patients with characteristics of metabolic syndrome. *Clin Ther* 2003; 25:1670–1686. 12860491 10.1016/s0149-2918(03)80162-5

[34] Steiner G Fenofibrate for cardiovascular disease prevention in metabolic syndrome and type 2 diabetes mellitus. *Am J Cardiol* 2008; 102 (12A):28L–33L. 10.1016/j.amjcard.2008.09.072 19084087

[35] Kraja AT Province MA Straka RJ Fenofibrate and metabolic syndrome. *Endocr Metab Immune Disord Drug Targets* 2010; 10:138–148. 20406163 10.2174/187153010791213047 PMC5278640

[36] Alexander CM Landsman PB Teutsch SM NCEP-defined metabolic syndrome, diabetes, and prevalence of coronary heart disease among NHANES III participants age 50 years and older. *Diabetes* 2003; 52:1210–1214. 12716754 10.2337/diabetes.52.5.1210

[37] Lakka HM Laaksonen DE Lakka TA The metabolic syndrome and total and cardiovascular disease mortality in middle-aged men. *JAMA* 2002; 288:2709–2716. 12460094 10.1001/jama.288.21.2709

[38] Noda H Iso H Saito I The impact of the metabolic syndrome and its components on the incidence of ischemic heart disease and stroke: the Japan public health center-based study. *Hypertens Res* 2009; 32:289–298. 19262490 10.1038/hr.2009.14

[39] Vishram JK Borglykke A Andreasen AH Impact of Age and Gender on the Prevalence and Prognostic Importance of the Metabolic Syndrome and Its Components in Europeans. The MORGAM Prospective Cohort Project. *PLoS One* 2014; 9:e107294. 25244618 10.1371/journal.pone.0107294 PMC4171109

[40] Jaffer F O’Donnell C Larson M Age and sex distribution of subclinical aortic atherosclerosis: a magnetic resonance imaging examination of the Framingham Heart Study. *Arterioscler Thromb Vasc Biol* 2002; 22:849. 12006401 10.1161/01.atv.0000012662.29622.00

[41] Carnethon M Bertoni A Shea S Racial/Ethnic differences in subclinical atherosclerosis among adults with diabetes: the multiethnic study of atherosclerosis. *Diabetes care* 2005; 28:2768. 16249554 10.2337/diacare.28.11.2768

[42] Bertoni AG Whitt-Glover MC Chung H The association between physical activity and subclinical atherosclerosis: the multi-ethnic study of atherosclerosis. *Am J Epidemiol* 2009; 169:444. 19075250 10.1093/aje/kwn350 PMC2726643

[43] Winston GJ Barr RG Carrasquillo O Sex and Racial/Ethnic Differences in Cardiovascular Disease Risk Factor Treatment and Control Among Individuals With Diabetes in the Multi-Ethnic Study of Atherosclerosis (MESA). *Diabetes Care* 2009; 32:1467. 19435957 10.2337/dc09-0260 PMC2713610

[44] Woo K Chook P Raitakari O Westernization of Chinese adults and increased subclinical atherosclerosis. *Arterioscler Thromb Vasc Biol* 1999; 19:2487. 10521379 10.1161/01.atv.19.10.2487

